# Advance directives in amyotrophic lateral sclerosis – a systematic review and meta-analysis

**DOI:** 10.1186/s12904-024-01524-1

**Published:** 2024-07-29

**Authors:** Anne Lisa Mangal, Martin Mücke, Roman Rolke, Iris Appelmann

**Affiliations:** 1 Department of Palliative Medicine, Medical Faculty, RWTH Aachen University, Pauwelsstraße 30, Aachen, 52074 Germany; 2 Department of Digitalization and General Practice, Medical Faculty, RWTH Aachen University, Aachen, Germany

**Keywords:** Amyotrophic lateral sclerosis (ALS), Motor neuron disease (MND), Advance care planning, Advance directives, Living will

## Abstract

**Background:**

Amyotrophic lateral sclerosis (ALS) is a neurodegenerative disease of the upper and lower motoneuron. It is associated with a life expectancy of 2–4 years after diagnosis. Individuals experience paralysis, dysphagia, respiratory failure and loss of communicative function, rendering advance care planning (ACP) critically important. This systematic review primarily aimed to internationally compare the application of advance directives (AD) and ACP in ALS. Its secondary aim was to identify ACP preferences, identify fields for future research and to generate recommendations for improving patient care through ACP.

**Methods:**

We conducted a systematic literature review and meta-analysis. Five electronic databases (Embase, Medline, Scopus, PsycInfo and CENTRAL) were searched for qualitative and quantitative primary literature from 1999 to 2024. Cross-references were used to identify additional publications. Study selection was performed based on inclusion criteria. Number and content of AD were extracted systematically. After statistical analysis consecutive meta-analysis was performed for international differences and changes over time. Quality assessment of studies was performed using the MMAT (Mixed Methods Appraisal Tool). PROSPERO Registration (June 07, 2021) : CRD42021248040.

**Results:**

A total of 998 records was screened of which 26 were included in the synthesis. An increase in publication numbers of 88.9% was observed from 1999 to 2024. Results regarding use and content of AD were heterogeneous and international differences were detected. AD were signed in 60.4% of records (1,629 / 2,696 patients). The number of AD decreased over time when separating the review period in two decades (1st 1999–2011: 78% vs. 2nd 2012–2024: 42%). Study quality was superior in qualitative and mixed method designs compared to quantitative studies.

**Conclusion:**

Further prospective studies should include detailed analyses on preferences regarding ventilation and artificial nutrition in ALS and should encompass countries of the global south. Despite the complexity of ACP with regard to individual patient needs, ACP should be part of each individual support plan for ALS patients and should specifically comprise a discussion on the preferred place of death. The available disease-specific AD documents should be preferred.

## Background

The term “advance care planning” (ACP) describes the process of planning one’s care preferences ahead. Advance directive (AD) documents are often synonymously called a “living will”. In this systematic review we refrain from using the latter term and exclusively refer to the “advance directives (documents)”.

ACP as a concept emerged in the United States from 1979 [1]. It is a process that supports the patient in identifying personal values and goals of care and deriving preferences for future medical care [2]. It often involves a series of consultations with a health care professional in which key points of the patients‘ wishes are discussed. The completion of an AD document may but must not necessarily be part of the ACP process [3].

An AD document can either be a standard form or written in the patients’ own words. The patient may list preferences regarding medical interventions or future care. This can include the wish to undergo certain procedures, for example the application of a feeding tube through the nose while in a state of unconsciousness. On the other hand it may convey the patient wish to forego certain interventions, such as an order of “do not resuscitate” (DNR) or “do not intubate” (DNI) [4]. Patients may also name a trusted person as a health care surrogate to express the patients’ wishes in case of fatal illness, either being a durable Power of Attorney for health care or the so called “health care proxy” [5].

Only one in three US adults has signed AD documents [6]. Furthermore, a qualitative study from 2020 shows that AD documents mostly consist of generic forms without disease specific modifications and are only very rarely revisited as physical and health changes occur [7].

Amyotrophic lateral sclerosis (ALS) is a neurodegenerative disease of central and peripheral parts of the motoneuron pathway leading to muscle paralysis, atrophy and spasticity [8–11]. Amyotrophic lateral sclerosis leads to an impediment of speech and swallowing [10]. This disease is an emblem of the experience of loss, not only of physical function but also of mental, social and emotional well-being so common in incurable neurological disease [9]. Peripheral skeletal muscles are often primarily affected, i.e. the small muscles of the hands and feet. This is inevitably followed by respiratory failure due to severe functional impairment of intercostal and other muscles critical for breathing [9, 10]. Disease trajectories can be classified primarily as spinal or bulbar, with a wide variety of other phenotypes getting recognized in the disease classification lately [11]. The incidence of ALS ranges from 0.6 to 3.8 / 100.000 and year, and prevalence is stated between 4.1 and 8.4 / 100.000, with both incidence and prevalence increasing [10–12]. The median survival time is between two and four years [11, 13, 14] while individual survival times differ vastly [10] ranging from months to up to ten years after diagnosis [9].

Given the rapid progression of muscle failure ventilator therapy significantly increases survival in ALS [15]. Different modes of ventilation such as non-invasive ventilation (NIV) via face mask or invasive ventilation (IV) via tracheostomy [16] may be applied to patients with ALS. Additionally artificial nutrition via nasogastric (NG) tube or percutaneous endoscopic gastrostomy (PEG) tube counteracts weight loss due to dysphagia, and these invasive measures may also prolong survival in ALS [17].

For ALS patients the feeling of burdening their caregivers adds to the disease burden itself [18]. While IV and NIV are associated with an overall adequate quality of life for the patients themselves, the burden for caregivers and next of kin is alleviated when a tracheostomy is in place [19]. The uncertainty of the disease trajectory and the remaining survival time adds to patient and caregiver burden. The ENCALS survival prediction model allows for a more personalized prognosis and may be used by a health care professional when initiating ACP in ALS [20]. Early and continuously revisited ACP is generally viewed as a key element when treating patients with ALS, considering the loss of communicative function, cognitive impairment and often rapid physical decline [9, 10]. Advance care planning has been incorporated in the European Federation of Neurological Societies (EFNS) guidelines on the clinical management of ALS [21] and the UK National Institute of Health and Care Excellence (NICE) evidence-based clinical guidelines on managing motor neuron disease [22].

Previous research in this field lacked rigor [23] and detailed analysis of patients’ wishes concerning supporting measurements near the end of life is scarce. More recent publications provide new insights into patient preferences for life- sustaining treatments.

Considering the low prevalence and incidence of ALS, publications dating back to 1999 reporting on the use of AD in ALS have been included in this review in order to assess the changes in AD use over time. This systematic review adds not only another decade of relevant literature since the systematic review published by Murray and Butow in 2016 [23] but also connects new findings through the meta-analysis, which will help identify trends regarding the global distribution of research and also to indicate a correlation between design and quality scores of the included studies.

This systematic review and meta-analysis aimed to present an international comparison of the application of ACP in ALS. Its secondary aims were to identify preferences regarding ACP in ALS, to uncover specific fields for future research and to ultimately derive recommendations for improving patient care.

## Methods

A systematic literature review and meta-analysis prospectively registered with PROSPERO (CRD42021248040) with the results reported according to the PRISMA guidelines for systematic reviews and later combined in a statistical meta-analysis [24].

### Inclusion criteria

Primary literature regarding ALS and the decisions made regarding end-of-life care and ACP were included. Studies concerning adults (age ≥ 18) were included in English and German language. Studies were only included if full texts were available. Conference abstracts were only included if sufficient data could be extracted. Qualitative and quantitative research published between 1999 and 2024 was included. Literature focusing on other neurological diseases but lacking subgroup analysis for ALS was excluded. Furthermore, literature concerning other terminally ill patients and their decisions near the end of life was excluded if not containing a subgroup analysis for ALS patients. Records exclusively dealing with quality of life in ALS and caregiver burden but lacking information on ACP or AD were also excluded. Literature reviews, systematic reviews and meta-analyses were also excluded. Lastly, editorials, commentaries and letters to the editor were excluded.

### Search strategy

Preliminary searches of the databases were undertaken from May 2020 until February 2023 and updated again in April 2024. The databases explored were Embase, Medline, Scopus, the Cochrane Central Register of Controlled Trials (CENTRAL) and PsycInfo. The search terms applied were (“amyotrophic lateral sclerosis” OR “motor neuron disease” OR mnd OR als ) AND ( “advance care planning” OR “advance directive” OR “end of life” OR “living will” OR Patientenverfügung OR Patientenverfuegung). In some cases, “NOT (cancer)” was added to specify the outcome. Search strategies were adapted to respective database guidelines.

In addition to the searches in these electronic databases, we also conducted reference searches in connected literature. Duplicates were manually removed from all identified records by the first reviewer (AM).

### Study selection

Selected studies were then screened against inclusion criteria via the abstract by AM. This process was repeated independently by the second reviewer (IA) when there was a lack of clarity on whether the record in question was suitable to be included. The remaining included records were then sought for retrieval and finally assessed for eligibility by the first and second reviewer.

Records not in agreement with inclusion criteria were excluded. Reasons for exclusion were lack of subgroup analysis for ALS, focus on care giver perspective and secondary record designs such as reviews and editorial letters. In some cases, records were eliminated from further review as they addressed the same study population. In these cases, the more relevant record was identified through the first (AM) and second reviewer (IA) and included in the review process. After the identification of full texts of the included records through electronic searches and correspondence with the primary authors, these manuscripts were screened again by both reviewers (AM and IA) independently.

### Data extraction

A data extraction sheet was created in Microsoft Excel^®^ (Version 2019) by the first and second reviewer (AM and IA). All numerical data was extracted for every record and charted accordingly. This was done by the first reviewer (AM) who consulted IA regularly when consensus was needed on whether data should be extracted. General information regarding author, year and country of publication was collected as well as the sample size and general attributes of the study population. The country of publication was defined by the location of the study population. Data regarding ACP and AD was extracted both regarding the existence and the content of the said directives within the study cohort. Fields of interest were among others timing and initiation of ACP and referral to life-prolonging measures in AD. These measures included mechanical ventilation (IV and NIV were extracted separately), tracheostomy and artificial nutrition via PEG or NG tube. For each item consent and refusal within the study cohorts were documented. Furthermore, we extracted data on the preferred place of death, the use of other AD documents (e.g. physician orders for life-sustaining treatment [POLST], health care proxy, Power of Attorney, DNR/DNI). Additionally, decisions made by the patients regarding end-of-life care directly without written AD were extracted separately. Data was collected by the first reviewer (AM) in Microsoft Excel^®^ and reviewed by the second reviewer (IA). Reporting was done in accordance with the Preferred Reporting Items for Systematic reviews and Meta-Analyses (PRISMA) 2020 Statement [24].

### Quality assessment

Quality assessment proved to be challenging as the literature consisted of qualitative as well as quantitative research. It was therefore performed using the Mixed Methods Appraisal Tool (MMAT, 2018 version) [25]. The MMAT allows for the critical assessment of methodological quality in different study designs when constructing a systematic literature review. For improved visualization, a ranking system was established ranging from 0 to 100% and was applied to all records included. The assessment was performed by the first reviewer (AM) and repeated by the second reviewer (IA) independently. Results were consistent in all but one case. After a verbal exchange between the two reviewers, consensus was reached for all studies included.

### Statistical analysis

Cataloging and management of retrieved records was performed with the citation management software EndNote™ Version X9 (Clarivate™, Philadelphia, Pennsylvania, USA). All relevant data was extracted from the identified records by the first reviewer. Health care proxies and signed Power of Attorney forms were not in all cases clearly distinguishable from other forms of AD and were therefore calculated separately.

In two cases the collected data referred to ALS patients but was retrieved through interviews with their caregivers or health care professionals only. Therefore, only the number of patients referred to was included in the cohort sizes for statistical analysis.

The number of patients in each cohort with AD documents was charted and calculated in relation to the respective cohort size. The random effects model was used to generate prevalence estimates. This was calculated with Meta XL Version 5.3 (EpiGear, QLD, Australia) in Microsoft Excel and visualized through a forest plot using GraphPad Prism^®^ 8 (GraphPad Prism Software, San Diego, California USA).

Two records containing information regarding AD in ALS and used for statistical analysis had to be excluded from the random effects analysis and forest plot. In one case the data was not numerical and in the other case the cohort size only referred to health care professionals being questioned about their ALS patients.

Mean difference and standard deviation were calculated for each item with Microsoft Excel^®^. Findings regarding international differences in the application of AD and the global distribution of publications were then analyzed further. Changes over time regarding the number of ALS patients in the cohorts who had signed AD documents were also calculated as relative figures for the consecutive meta-analysis. For a more intuitive visualization of the presented results and in order to address the detected heterogeneity, we decided to split the review period into two decades, the first beginning in 1999 and ending in 2011 and the second beginning in 2012 and ending in 2024. This proceeding allows for a more efficient comparison of the existing data cohorts over time. This is supported by two additional forest plots presenting the subgroup analysis of these two decades respectively. All three forest plots show the prevalence estimates and their confidence intervals, the weighted percentage of the individual cohorts in proportion to the combined study cohorts and *I*^*2*^ as a measure of heterogeneity.

Scores of the MMAT assessment tool for study quality were analyzed together with the distribution of study designs and respective MMAT scores for each design. For this a one-way ANOVA was performed using GraphPad Prism^®^ 8. Figures and illustrations were created using GraphPad Prism^®^ 8 and Microsoft Power Point^®^ (Version 2019).

## Results

### Study selection

**Fig. 1:**
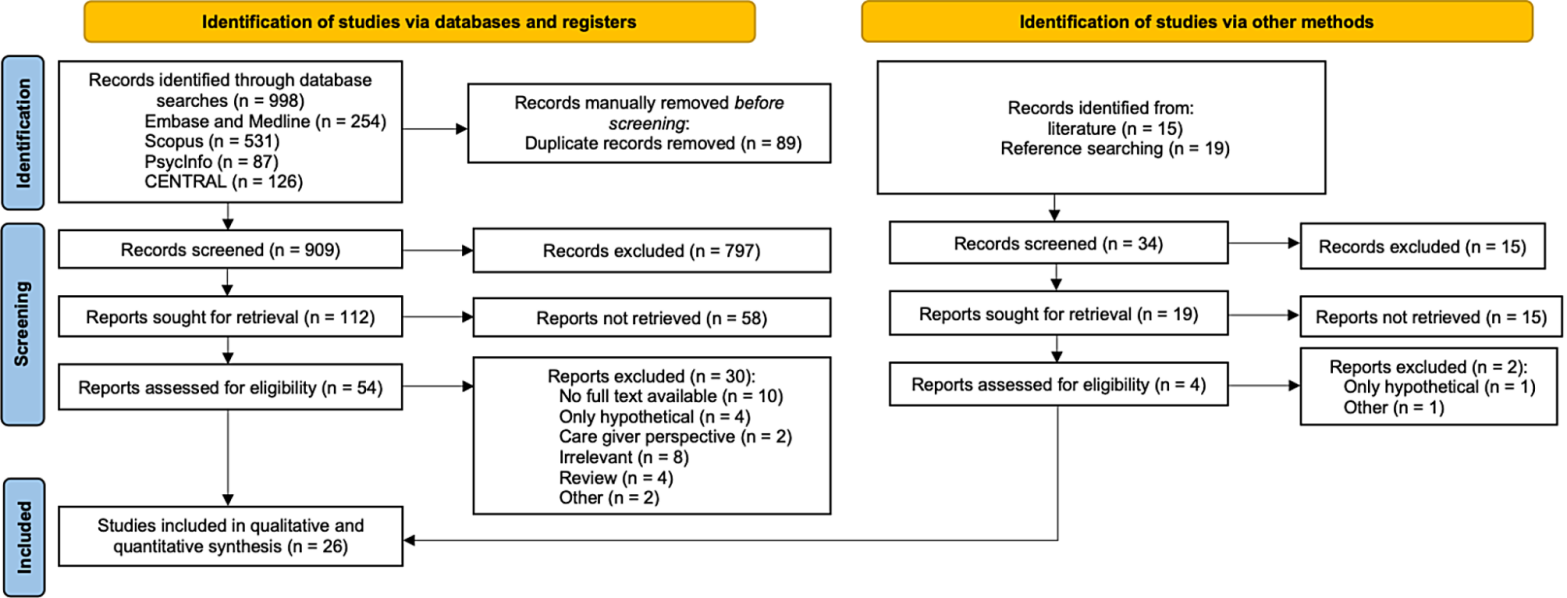
PRISMA Flowchart (2020 version) [24]

Initial searches generated 998 records. After the removal of duplicates and the first screening of titles and abstracts, 112 records were left for retrieval. Twelve corresponding authors were contacted to identify missing full texts. Fifty-four records were then assessed for eligibility. One author group was still waiting for publication of their study which could therefore not be included. One record had to be excluded subsequently as the same patient cohort was analyzed in two publications by the same research team but published by different primary authors. The preliminary figure of 22 records identified in 2020 through electronic database searches was enlarged by two records identified through reference searches of corresponding literature and two additional records identified through updated electronic database searches in April 2024. The final synthesis and meta-analysis therefore consists of 26 records. The selection process is visualized in more detail in the PRISMA Flowchart (Fig. 1) [24].

### Study characteristics

**Fig. 2:**
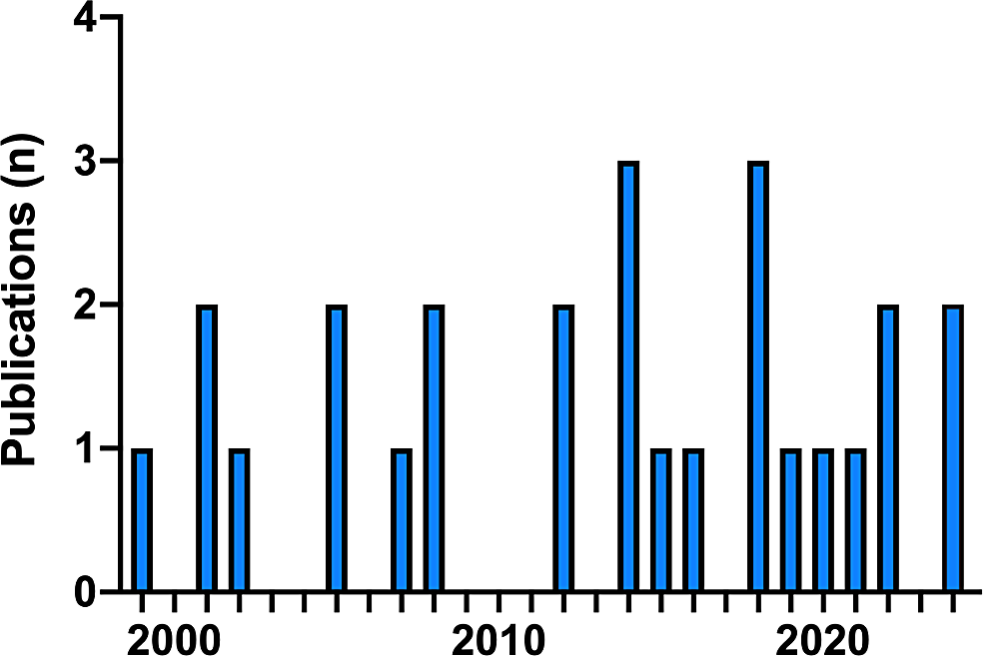
Years of publication. Bar diagram illustrating the total number of publications per year over the review period from 1999 until 2024. The diagram shows an increase of 88.9% when comparing the first with the second decade

The 26 included studies were published between 1999 and 2024. Between the year 1999 and 2011 only nine studies were identified [26–34] but there was an 88.9% increase in publications over the next 12 years, with 17 studies published [35–51] (Fig. 2and Table 1).

**Table 1:** Included publications and key characteristics

Author, Year	Study Design	Sample size, Subject	Method	Results	Quality (%)
Vandenbogaerde et al. [51]	Qualitative: longitudinal grounded theory	9 ALS patients and their care givers	Three interviews over 9-month period regarding Advance care planning (ACP)	All patients thought about ACP but not all talked about it, personal factors influence decision-making	100
Phillips et al. [50]	Quantitative: descriptive survey	49 ALS patients assisted by their care givers	Questionnaires regarding ACP and advance directives (AD)	Patients feel well informed, no correlation between time of diagnosis and ACP	100
Klock et al. [48]	Quantitative: Non-randomized analytic cross-sectional	130 ALS patients	Retrospective chart review of health care utilization of patients with and without AD	No difference in health care utilization but more palliative care (PC) consultations after ACP discussions	60
Takacs & Comer [49]	Quantitative: Non-randomized analytic cross-sectional	513 ALS patients	Retrospective chart review to assess number and content of AD	Only 1/3 had AD, very few had Physician Orders for Life Sustaining Treatment (POLST) or saw PC consultant	80
Sukockiène et al. [47]	Quantitative: Non-randomized Cohort	64 ALS patients	Retrospective chart review of when and how ACP is initiated after implementing PC consultant in multi-professional team	ACP can be discussed at first visit to PC consultant, completion rates are high	100
Mehta et al. [46]	Quantitative: Non-randomized analytic cross-sectional	28 ALS patients	Retrospective chart review of ALS patients with and without PC consultation	PC consultation leads to earlier and more thorough ACP	80
Seeber et al. [45]	Qualitative: Grounded theory	21 ALS patients	Observational interviews and in-depth analysis of ACP conversations	ACP is a process	100
Cheng et al. [42]	Quantitative: Non-randomized analytic cross-sectional	42 ALS patients	Patients were referred to PC specialist to assess PC needs	Need for early PC consultation	80
Kettemann et al. [43]	Quantitative: Non-randomized Cohort	102 ALS patients	Evaluation of ACP concept regarding withholding and withdrawing of life prolonging measures through interviews	High acceptance of ACP, treatment in accordance with patients wishes	60
Moglia et al. [44]	Quantitative: Descriptive cross-sectional	452 ALS patients	Retrospective chart review of ALS patients to assess role of AD	Gender and age does not affect ACP, correlation between nasogastric (NG) Tube and foregoing of tracheostomy	40
Murray et al. [41]	Qualitative: Narrative research	18 Care givers	Semi-structured interviews with care givers of ALS patients on Letters of future care (LFC)	LFC completion was beneficial for care givers and ALS patients themselves	100
Chhetri et al. [40]	Quantitative: Non-randomized analytic cross-sectional	99 ALS patients	Retrospective chart review regarding place of death and Preferred Priorities for Care (PPC) document	Most ALS patients would prefer to die at home	60
Lulé et al. [37]	Quantitative: Non-randomized Cohort	93 ALS patients	Three follow up interviews over one year regarding Quality of Life (QoL) and attitude towards hastening death	More positive attitude towards health care interventions (Non-invasive-ventilation (NIV) / Percutaneous endoscopic gastrostomy (PEG) / invasive ventilation (IV)) towards end of life	80
Maessen et al. [39]	Quantitative: Non-randomized Cohort	102 ALS patients	Questionnaires every 3 months about QoL and physician assisted suicide (PAS)	Wish for PAS does not lead to lesser care or loss of QoL in ALS patients	100
Martin et al. [38]	Quantitative: Non-randomized Cohort	78 Care givers and ALS patients	Interviews at various disease stages but always after having made a treatment decision	Treatment decisions may not be influenced by illness trajectory but also by personal characteristics	60
McKim et al. [35]	Quantitative: Non-randomized Cohort	26 Care givers and ALS patients	Interviews before and after educational intervention regarding NIV and IV	Significant improvement in knowledge, projection of future ventilator choices	80
Stutzki et al. [36]	Quantitative: Descriptive survey	33 care givers and ALS patients	Questionnaires and Interviews about PAS and life prolonging measures	ALS patients are more against NIV and PEG than care givers, Legality does not promote wish for PAS	80
Kühnlein et al. [33]	Mixed method: Convergent design	29 Care givers	Structured interviews with care givers of deceases ALS patients about thoughts towards PAS	Only few patients in Germany seem to have thoughts about PAS	80
Nolan et al. [34]	Mixed method: Sequential explanatory design	32 Care givers and ALS patients	Questionnaires and semi-structured interviews regarding patients` wish to include care givers in decision-making process	Patients who preferred shared decision-making ended up making decisions more independently than they had hoped	100
Munroe et al. [32]	Quantitative: Descriptive cross-sectional	42 ALS patients	Retrospective chart review of end of life preferences among ALS patients	Decisions regarding medical treatment are often delayed	80
Burchardi et al. [31]	Qualitative: Grounded theory	30 ALS patients and clinicians	Interviews with patients and clinicians about discussing AD	Wait-and-see-policy	100
Pautex et al. [30]	Quantitative: Descriptive cross-sectional	22 ALS patients	Retrospective chart review to detect discrepancies between recommendations and clinical practice	Discrepancies between recommendations and clinical practice were detected	60
Ganzini et al. [29]	Quantitative: Descriptive survey	50 Care givers	Retrospective chart reviews and interviews with care givers about final months of ALS patient	Patients still experience destress despite enrollment with PC specialist	100
Borasio et al. [27]	Quantitative: Descriptive survey	73 Clinicians	Clinicians all over Europe answered questionnaire regarding treatment of ALS patients in different disease stages	Need for uniform European standard in care of ALS patients	40
Mandler et al. [28]	Quantitative: Descriptive survey	1014 Care givers	Retrospective chart reviews and interviews with care givers of deceased about end of life care	Terminal care was overall well managed, care givers feel there is room for improvement	40
Albert et al. [26]	Quantitative: Non-randomized Cohort	118 ALS patients	Baseline and follow up-interviews every 4 months, prospective assessment of PC use	Comparable use of PC over disease trajectory but rising number of tracheostomies over time	100

The included literature is presented chronologically, sorted by the year of publication. The quality assessment was conducted using the Mixed Methods Appraisal Tool and ranged from 0 to 100%

Of the 26 included studies, the majority (76.9%, *n* = 20) were quantitative studies. A smaller number were mixed methods (*n* = 2), or qualitative (*n* = 4) studies. Of the qualitative studies, two utilized grounded theory [31, 45], one was narrative research [41] and another a longitudinal qualitative study with a grounded theory approach [51].

Of the two mixed methods studies one used a convergent design [33] while the other utilized a sequential explanatory design [34].

**Fig. 3:**
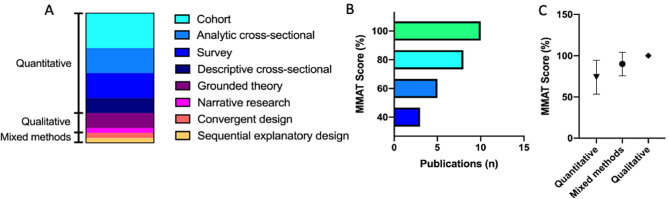
Association of study design and MMAT Score. **A**: 77% were quantitative studies, 15.4% qualitative studies and 7.7% were mixed methods study designs. **B**: The Mixed Methods Appraisal Tool for the assessment of study quality in its 2018 version was applied. For better visualization, a scoring system ranging from 0 to 100% was applied to all included records. **C**: One-way ANOVA was performed in GraphPad Prism 8. Symbols representing means and error bars SD

Among the quantitative studies were three descriptive cross-sectional studies, five analytical cross-sectional studies, five surveys and seven non-randomized cohort studies (Fig. 3A).

Most studies (42.3%, *n* = 11) employed a retrospective medical file review and 38.5% personal interviews, while 34.6% chose questionnaires.

Overall the qualitative and mixed methods studies had higher quality scores than the quantitative studies. Only a third (38.5%, *n* = 10) of the studies had a high quality score of 100% and eight studies (30.7%) had a quality score of 80%. Five studies (19.2%) were assessed with a quality of 60% and three studies (11.5%) were assessed with a quality of 40% (Fig. 3B). All four qualitative studies [31, 41, 45, 51] received a quality score of 100% and the two mixed methods approaches [33, 34] 80% and 100% respectively (Fig. 3C). In 30% of the quantitative studies quality score was impacted by the lack of acknowledgement of confounders or inclusion and exclusion criteria leading to a 20% reduction in the quality assessment score.

**Fig. 4:**
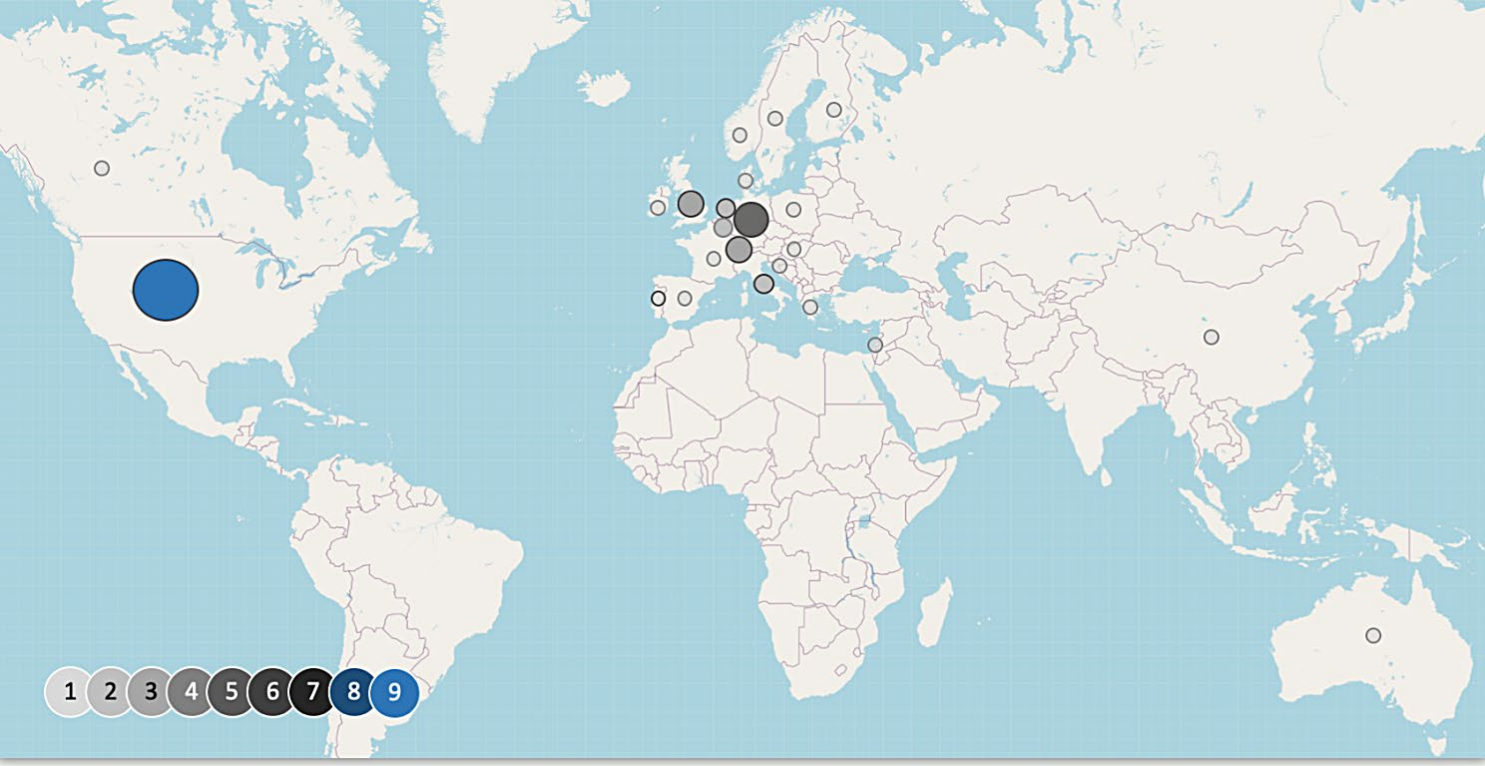
Distribution of participating countries. World map indicating the location of origin for the included records. The circle size represents a number of publications in the respective country and shows the focus of research in countries of the global north. © OpenStreetMap

The studies were undertaken in 23 different countries, with most occurring in the United States of America (USA) (*n* = 9) [26, 28, 29, 32, 34, 46, 48–50] or Germany (*n* = 5) [27, 31, 33, 37, 43]. A smaller number took place in Switzerland (*n* = 3) [30, 36, 47] or the United Kingdom (*n* = 3) [27, 38, 40]. Other studies included participants from Italy [27, 44], Belgium [27, 51] and the Netherlands [39, 45], respectively. Seventeen additional countries were included largely as part of Borasio et al. [27] 2021 international survey involving participants from 14 countries (Fig. 4).

### Study population

The cohort size varied from *n* = 15 [31] to *n* = 1014 participants [28]. In 15 studies (57.7%) only the ALS patient was interviewed [26, 30, 32, 37, 39, 40, 42–50]. In one study (3.8%) only health care professionals were interviewed [27] and in four publications (15.4%) the respective caregiver [28, 29, 33, 41]. In five publications (19.2%) data was analyzed from patients and caregivers [34–36, 38, 51] and in one case (3.8%) from patients and health care professionals [31]. In four cases the study cohort consisted of an equal number of patients and caregivers [34–36, 51] and in one case of patients and neurologists [31]. The sex of the participants was documented in 19 studies (73.1%), and they were either classified as male (57%) or female (43%). Any information on gender was not included in the analyzed studies. Religion and spirituality of the patients were documented in five studies (19.2%) [27, 33, 36, 38, 50].

### Advance care planning

Eleven out of 26 included studies (42.3%) contained information regarding ACP discussions with ALS patients. In seven additional publications it was unclear whether the signing of AD documents was preceded by ACP conversations. Six studies (23.1%) contained numeral data on the number of patients in the cohorts, that had ACP communication with their health care professional. Two hundred forty-nine out of 341 patients in the respective cohorts (73%) were documented to have had structured conversations about end of life wishes and care goals.

Advance care planning was mostly addressed by the physician (in 63.6% of studies) rather than by the ALS patients themselves. Three German publications stated the topic of ACP being risen by the physician first while only one German study found the patient to seek exchange regarding this subject first.

Eight out of 26 publications (30.8%) stated the time after diagnosis when ACP was first initiated with the patient. In six out of these eight reports (75%) it was initiated early after diagnosis. Three publications (37.5% out of eight publications) specified this to be at the first visit after diagnosis [32, 46, 47], which was the first consultation with a palliative care (PC) specialist, a hospital admission or a consultation with another health care professional. In one report, ACP was incorporated in the conveying of the diagnosis [27]. In two out of eight publications (25%) ACP was postponed until symptoms occurred, or the patients’ physical condition worsened.

### Advance directives

Nineteen studies (73.1%) detailed patients’ preferences regarding AD. The fraction of ALS patients that had signed orders regarding care or treatment preferences ranged from 9.1% (2/22 patients) [30] to 90.5% (19/21 patients) [45] with a median of 60.4% (1,629/ 2,696 patients). Figure 6A visualizes this heterogeneity with I^2^ = 98%. At the same time, the plot shows that the smaller cohort sizes contribute equally to the AD results within the group of ALS patients as the weighted proportions are all within a similar range.

Healthcare proxies and signed Power of Attorney forms were described in five reports. 25.2% of patients (175/ 695 patients) in these studies had a healthcare proxy, and 20.8% (145/ 697 patients) had signed Power of Attorney forms.

Patients’ preferences for IV therapy were documented in two studies, with 6.2% of the patients in favour of (2/32 patients with AD [32]) and 94.4% declining IV therapy (17/18 patients with AD [33]). Patient preferences regarding non-oral nutrition were only documented in one study (Takacs and Comer [49]). A total of 74 (48%) of the 154 ALS patients in the cohort that had signed AD had included PEG or NG tube placement in their advance care documentation, and 34 out of 74 patients (46%) were against and 40 out of 74 patients (54%) were in favor of the intervention. No detailed analysis of written preferences for NIV was documented in any of the reviewed studies.

International discrepancies both regarding the number of signed AD and their contents could be detected. In the USA 63.5% of 1,960 patients in the study cohorts had signed AD, while in Germany it was 55.6% of 117 ALS patients. In Switzerland only 36.4% of 86 patients in the study cohorts had signed AD.

Whether or not the ALS patients in the cohorts used a standard AD form was only documented in two reports [31, 46]. Therefore, the 95.2% of patients that used a standard form to document their preferences represents 20 out of 21 patients in the cohorts in question.

Six records described patients’ use of DNR/DNI forms and 15.1% of the evaluated patients (122 out of 807 patients) had a DNR/DNI form signed.

In four cases other specific documents were set up, one of them being the Preferred Priorities for Care (PPC) form. This document enables the patient to express their wishes about the preferred place of death. Among the 53% of patients that made use of the PPC form half (56%) named home as their preferred place of death, with a smaller number preferring hospice (19%) and only a few nominating a nursing home (*n* = 4). None chose a hospital.

**Fig. 5:**
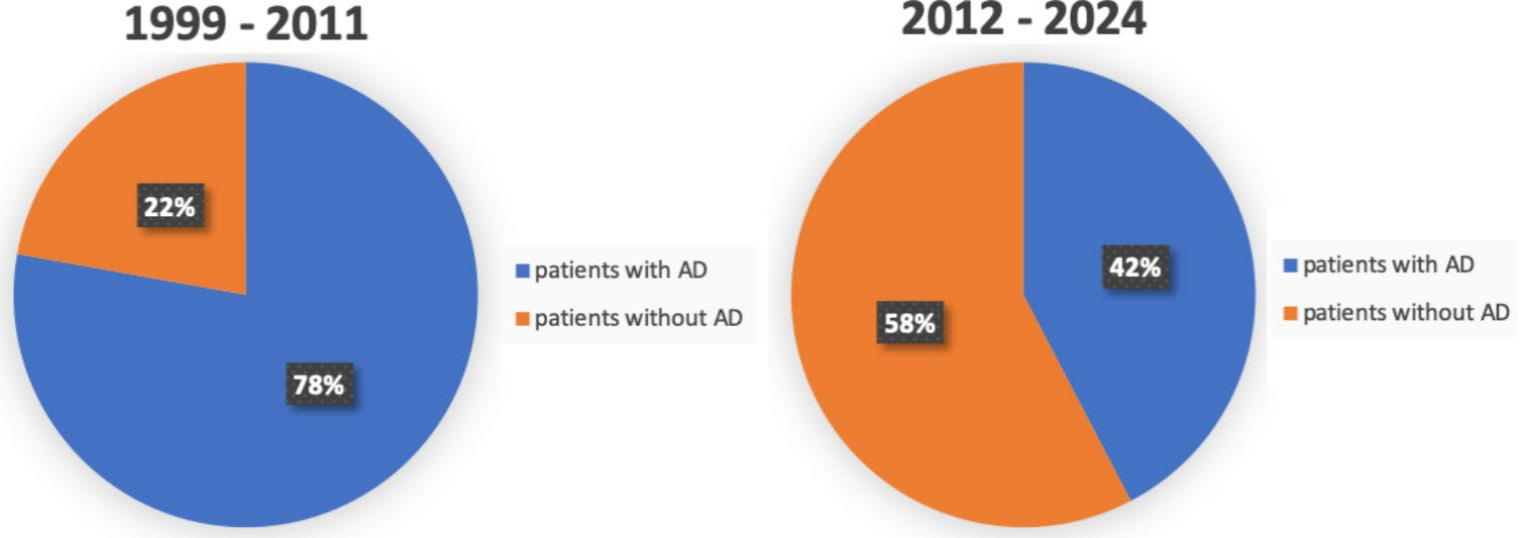
Use of advance directives among ALS patients over time. Diagram illustrating the use of advance directives (AD) among ALS patients over the review period. Left: 1st review decade from 1999 until 2011. Right: 2nd decade from 2012 until 2024. Significant decrease of patients with signed AD from 78 to 42% in the more recent literature

**Fig. 6:**
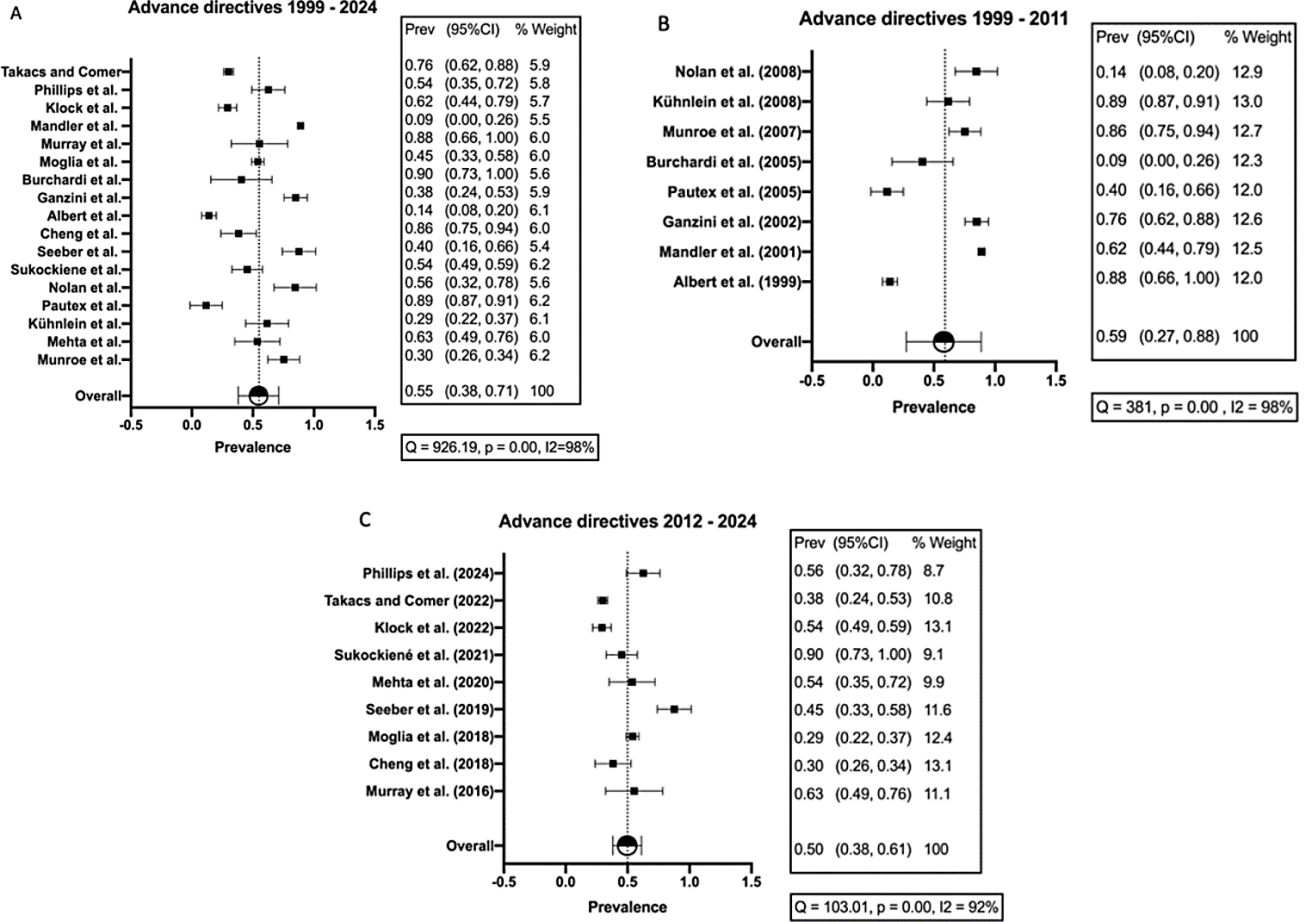
Forest plots for prevalence estimates on advance directives (AD) in ALS over time. Forest plots representing prevalence estimates over time with 95% CI, % Weight and Heterogeneity analysis (I2). **A**: Prevalence estimates over complete review period. Overall estimate is 55%. Heterogeneity estimate I ^2^ = 98%. **B**: Prevalence estimates in first decade (1999–2011). Overall estimate is 59%. Heterogeneity estimate I^2^ = 98%. **C**: Prevalence estimates in second decade (2012–2024). Overall estimate is 50%. Heterogeneity estimate I^2^ = 92%. The prevalence estimate is slightly higher in first compared to second decade. Heterogeneity significantly elevated in all analyses

When comparing the use of AD over the review period, we detected a decline even though the combined study cohorts for the first and the second review decade remain relatively stable (1,379 and 1,317 study participants respectively). In the first decade from 1999 to 2011, 77.8% (1073 out of 1379 patients) had signed documents. Studies published between the year 2012 and 2024 however show that 42.2% (556 out of 1317 patients) had signed AD (Fig. 5). The prevalence estimate we calculated as a subgroup analysis for these two decades is presented in Fig. 6B and C. Shown is an estimated prevalence of 59% in the first and of 50% in the second decade. Additionally, the heterogeneity estimate I^2^ was at 98% in the first and at 92% in the second decade respectively, supporting our previous findings of I^2^ = 98% in the overall study cohorts. The existing data was not sufficient to measure a possible shift in the preference or use of certain medical interventions among the ALS patients in the cohort.

### Decision-making regarding medical measures

Sixteen studies (61.5%) reported a mean of 59.2% of ALS patients (232/ 392 patients) who made a therapeutic decision near the end-of-life. In twelve studies, a decision regarding ventilation was made. While an average of 8% of patients (22/ 274 patients) chose IV, 46.9% (134/ 286 patients) refused this measure. NIV was elected in 46.8.% of cases (108 out of 231 patients) and rejected in 11.4% (24 out of 299 patients) in the cohort.

In eight cases patients’ decisions concerning the placement of a nasogastric feeding tube were examined. While 18.7% (65 out of 348 patients) chose to abstain from a tube placement, 23.7% (75 out of 317 patients) decided in favor of the intervention.

Two studies discussed patients’ decision to end therapeutic measures that had been implemented in the past [29, 43]. 6.7% (*9 out of 135 patients*) decided to remove a feeding tube, that had been placed earlier. 12.3% (15 out of 122 patients) chose to discontinue IV while 1% (one out of 102 patients) chose to terminate NIV.

## Discussion

### Study characteristics

The Thapar University in Patiala, India conducted a bibliometric assessment in 2016 on global research trends from 1976 until 2013 regarding ALS. Ram et al. found an annual growth rate of 39.6% of ALS related research with no clear shift of research focus [52]. This supports our findings, that the number of relevant publications regarding AD and ACP in ALS has grown rapidly since 1999 and indicates a growing awareness for ALS among the medical research community.

Shared decision-making has been shown to improve patient satisfaction [53] and is increasingly implemented in clinical practice guidelines [54, 55]. Kilbride and Joffe call this ‘the new age of patient autonomy’ [56]. This might contribute to the increased interest in AD and ACP in patients with terminal and life limiting diseases such as ALS. This research trend, however, it contrasts with a decline in AD frequency in the studied ALS population.

Notably, all included qualitative [31, 41, 45, 51] and mixed methods publications [33, 34] attained a quality score of 80% or higher according to the MMAT [25]. This might be a result of more elaborate study designs and may indicate the necessity for more qualitative and mixed methods approaches in this field of research. Another explanation is the different assessment approaches within the MMAT for different study methods. Lack of confounder acknowledgement and missing transparency concerning inclusion and exclusion criteria result in reduced quality scores in 60% of quantitative studies with scores of 80% or lower. Both these criteria were not part of the quality assessment of the MMAT for qualitative and mixed method study designs. This may lead to decreased comparability among the assessed publications.

Not a single randomized controlled trial was included in this systematic review, strongly indicating the future need for such studies.

Another prominent aspect is the divergence within the countries of publication. 34.6% of included studies focus on patients with ALS in the USA and 19.2% represent cohorts of German ALS patients. This could be interpreted as an inclusion-criteria and selection-process bias as only publications in German and English were included.

### Study population

Cohort sizes differed remarkably between publications. All included qualitative studies show relatively smaller cohort sizes. This is justifiable through a more complex mode of data collection and analysis. While some researchers conducted quantitative studies with hundred or more participants, this seems to be the exception rather than the rule. This is possibly on account of the low incidence of ALS among the general population [12]. According to Leighton et al. local rises in ALS incidence in Scotland are most likely to be caused by improved neurological coverage and not a sign of a rising incidence globally [57].

In most cases patient data was retrieved from local ALS registries or ALS research centers, which allows for a more representative cohort size in a disease with such low incidence and prevalence. Documentation of personal factors influencing the decision-making process was missing in most studies. Spirituality as one impact factor in the process of decision-making was also rarely documented. Strikingly, none of the included publications acknowledged this as a possible confounder.

### ACP

The topic of ACP is mainly addressed by the health care professional. In Burchardi et al. patients consider limiting medical treatments to be something offending their physician as they are bound by an oath. Seeber et al. found, that ALS patients preferred less information on treatment options and medical interventions [45]. This is in line with our findings, that in 28.6% of publications, ACP was postponed until the patients’ felt their condition worsened. This agreed with both, the patients’ and the physicians‘ wishes in these reports, as both groups felt, conversations about medical interventions would imply impending death [31]. Pautex et al. on the other hand state that some patients felt ill-informed on the treatment options and had been expecting their physician to address ACP [30]. In most included publications, the early commencement of ACP is part of standard procedure.

It is surprising that such little consideration is given to ACP in ALS in current literature especially as it is a key element of several international best practice guidelines regarding the management of patients with ALS [21, 22].

### Advance directives

Complementary to patients’ direct decision to forego IV, it was also widely rejected in the documented AD. Most ALS patients chose to communicate their wishes through a standard form. This practice leads to a binary decision-making that does not necessarily reflect patients’ individual preferences for future medical interventions.

The evaluation of the PPC documents showed that none of the ALS patients prefer to spend their last hours in a hospital. According to Klock et al., however, 10% of ALS patients in the study cohort die in hospitals regardless of signed AD or ACP discussion with their physician [48]. A possible explanation for this discrepancy might be that first responders - if called to the case of an unconscious ALS patient - are not informed of the patients’ wishes and therefore opt for transportation to a hospital. Also, the distress that the worsening condition of the disease causes for the caregivers [36] could lead to premature institutionalization.

According to Burchardi et al., ALS patients feel signing an AD is proof of their oncoming death and are therefore hesitant to raise this issue with their physician [31]. The latter on the other hand are often reluctant to discuss AD with their patients for the same reason. This is described by Burchardi et al. [31] as the ‘wait-and-see-policy’ that might contribute to the fluctuating number of ALS patients with documented AD.

Our results show a decrease in the use of AD over the review period when comparing the subgroup of the first with the second decade. This seems even more apparent when comparing the actual number of patients in the cohorts who had signed AD documents considering the comparable sizes of both subgroups. The relevant decrease in the prevalence of AD in ALS patients over time might be partly explained due to the SARS CoV2 pandemic and hence decreased frequency of especially face-to-face physician-patient contacts. The significant heterogeneity in all three prevalence estimates is an important finding that describes the unmet need of better and timely clinical communication with ALS patients regarding this topic. This also suggests the need for more clinical research in this context to minimize barriers to the creation of AD and facilitate their use in ALS.

### Decision-making regarding medical measures

Most patients in the reviewed publications chose to forego IV while NIV was widely accepted. This is in line with a scoping review conducted in 2022, where 23% of ALS patients chose IV, 35% chose NIV and 42% chose both at some point during their illness [16].

Our findings show that IV therapy was also more often discontinued than non-invasive options. The two publications presenting these results represent a study cohort based in the USA (Ganzini et al. [29]) and Germany (Kettemann at al. [43]). In Germany the patient’s wish to end therapeutic measures is to be honored and protected by law [58]. In the United States the case of Schloendorff vs. the Society of New York Hospital in 1914 established precedence [59]. Continuing therapy against the explicit will of the patient constitutes medical battery and is punishable by law.

The decision for or against a NG tube placement was slightly more balanced. According to Labra et al., the reasons for either decision are often linked to their personal surroundings. ALS patients tend to feel like a burden to their caregivers [17]. They hope to either alleviate this burden by adding artificial nutrition and therefore being able to contribute more to their family’s everyday lives. On the other hand, by foregoing artificial nutrition, they expect to shorten the duration of their disease and therefore limit care giver burden [17].

Martin et al. concluded in a prospective population study, that ALS patients were more likely to forego medical interventions if they were employed at the time of their diagnosis [38]. Also, fewer depressive symptoms were associated with a higher rate of refusal of said interventions.

### Limitations/strengths

A possible impediment of this systematic review is the limited number of relevant publications that were included after the selection process and the heterogeneity concerning the prevalence of ACP. This made it inevitable to include not only very recent publications but also earlier ones, the contents of which might be partly outdated. Also, the inclusion of publications in English and German only may limit an international comparison.

The signing of an AD document was not always preceded by structured ACP conversations. The lack of a clear distinction between the two within the included literature further obstructed our analysis.

The number of ALS patients within the cohort who had signed AD differed vastly between publications. This emphasizes the need for a strategic and systematic evaluation of the existing data. Conducting a meta-analysis accentuates the relevance of the presented results and constitutes a strength of this present work.

## Conclusion

### Future fields of research

As the general interest in ALS appears to be growing, more prospective research in the field should be conducted. Especially supplementary information on documented decisions regarding NIV and NG-tube placement is required. Future research should include qualitative and quantitative study designs as this allows for a more differentiated view on AD in ALS. This is also relevant when determining whether quantitative research in this field generally tends to lack rigor when compared to qualitative and mixed methods approaches. Furthermore, the focus of publications in the countries of the global north generates an unbalanced representation of data. Inclusion of research in languages other than English and German could lead to a more complete understanding of ALS patients needs and their living realities.

Possible confounders to the decision-making process near the end of life such as spirituality or familiar background should also be considered when pursuing this field of research.

As not a single relevant controlled trial regarding the use of ACP and AD in ALS was identified, future research approaches should aim to include this level of scientific evidence to consolidate and broaden the current state of research.

### Clinical recommendations

ACP should be part of an individual support plan for each ALS patient. Our results show that patients’ expectations towards their physician in this regard vary greatly. Therefore, timing and scope of ACP should be individually discussed and adapted to patient preferences. A conversation offer should be made by the physician early on after conveying the ALS diagnosis and regular reevaluation of therapeutic goals and consecutive needs is certainly indicated. This way the patient has time to reflect ones wishes and discuss treatment options with their physicians over the course of the disease if the need should arise.

According to our research, ALS patients’ preferred place of death is in some cases not in line with their expressed wishes. This should therefore be part of regular communication between the physician and the ALS patient.

The presented results about the decisions ALS patients make regarding medical measures are overall heterogeneous. To deduce recommendations based on the existing data was therefore challenging. Nonetheless it is our impression, that the medical choices the patients in the cohorts made, were often dependent on their personal surroundings, familiar support systems and overall education through their physicians. NG Tube placement and NIV should be addressed by the treating physicians at an early stage of ALS. This permits the patient, to investigate the various treatment options at length and decide in advance. Practical demonstrations of different modes of ventilation as proposed by McKim et al. [35] could facilitate the decision-making process for patients and care givers.

Standard forms for AD although widely used do not offer the ALS patient the necessary differentiation. Disease specific forms should therefore constitute a new standard for ALS patients. Those are exemplarily provided by Benditt et al. [60] in English and by the ALS outpatient department of the University Hospital Charité in Berlin in German [61] .

We conclude that ALS patients dealing with the issue of drafting an AD document should consider all fields of medical interventions relevant for their disease trajectory. Especially the decisions regarding ventilator options and artificial nutrition should be a key element of an AD in ALS. Choices made regarding their preferred place of death should also be put in writing and be respected if reasonable from a medical point of view.

The decreasing number of ALS patients with AD documents should be counteracted by an empathetic and repeated offer for dialogue between the ALS patient and the health care professional. The described heterogeneity of data on the prevalence of AD in ALS is a clear indicator of the unmet need to raise awareness of timely conversations about end-of-life preferences.

## Data Availability

A full table of all screened articles primarily included in this systematic literature review can be obtained from the corresponding author.

## Abbreviations

ACP: Advance care planning
AD: Advance directive
ALS: Amyotrophic lateral sclerosis
CENTRAL: Cochrane Central Register of Controlled Trials
DNI: Do not intubate
DNR: Do not reanimate / resuscitate
IV: Invasive ventilation
MMAT: Mixed Methods Appraisal tool
NG: Nasogastric (tube)
NIV: Non-invasive ventilation
PEG: Percutaneous endoscopic gastrostomy
PC: Palliative Care
PPC: Preferred Priorities for Care Document
POLST: Physician Orders for Life Sustaining Treatment
USA: United States of America
